# Proteomics analysis of high lipid-producing strain *Mucor circinelloides* WJ11: an explanation for the mechanism of lipid accumulation at the proteomic level

**DOI:** 10.1186/s12934-016-0428-4

**Published:** 2016-02-11

**Authors:** Xin Tang, Xinyi Zan, Lina Zhao, Haiqin Chen, Yong Q. Chen, Wei Chen, Yuanda Song, Colin Ratledge

**Affiliations:** State Key Laboratory of Food Science and Technology, School of Food Science and Technology, Jiangnan University, Wuxi, People’s Republic of China; Colin Ratledge Center for Microbial Lipids, School of Agriculture Engineering and Food Science, Shandong University of Technology, Zibo, People’s Republic of China; Synergistic Innovation Center for Food Safety and Nutrition, Wuxi, People’s Republic of China; Department of Biological Sciences, University of Hull, Hull, UK

**Keywords:** Lipid accumulation, *Mucor circinelloides*, Nitrogen deficiency, Proteomics

## Abstract

**Background:**

The oleaginous fungus, *Mucor circinelloides*, is attracting considerable interest as it produces oil rich in γ-linolenic acid. Nitrogen (N) deficiency is a common strategy to trigger the lipid accumulation in oleaginous microorganisms. Although a simple pathway from N depletion in the medium to lipid accumulation has been elucidated at the enzymatic level, global changes at protein levels upon N depletion have not been investigated. In this study, we have systematically analyzed the changes at the levels of protein expression in *M. circinelloides* WJ11, a high lipid-producing strain (36 %, lipid/cell dry weight), during lipid accumulation.

**Results:**

Proteomic analysis demonstrated that N depletion increased the expression of glutamine synthetase, involved in ammonia assimilation, for the supply of cellular nitrogen but decreased the metabolism of amino acids. Upon N deficiency, many proteins (e.g., fructose-bisphosphate aldolase, glyceraldehyde-3-phosphate dehydrogenase, enolase, pyruvate kinase) involved in glycolytic pathway were up-regulated while proteins involved in the tricarboxylic acid cycle (e.g., isocitrate dehydrogenase, succinyl-CoA ligase, succinate dehydrogenase, fumarate hydratase) were down-regulated, indicating this activity was retarded thereby leading to a greater flux of carbon into fatty acid biosynthesis. Moreover, glucose-6-phosphate dehydrogenase, transaldolase and transketolase, which participate in the pentose phosphate pathway, were up-regulated, leading to the increased production of NADPH, the reducing power for fatty acid biosynthesis. Furthermore, protein and nucleic acid metabolism were down-regulated and some proteins involved in energy metabolism, signal transduction, molecular chaperone and redox homeostasis were up-regulated upon N depletion, which may be the cellular response to the stress produced by the onset of N deficiency.

**Conclusion:**

N limitation increased those expressions of the proteins involved in ammonia assimilation but decreased that involved in the biosynthesis of amino acids. Upon N deprivation, the glycolytic pathway was up-regulated, while the activity of the tricarboxylic acid cycle was retarded, thus, leading more carbon flux to fatty acid biosynthesis. Moreover, the pentose phosphate pathway was up-regulated, then this would increase the production of NADPH. Together, coordinated regulation of central carbon metabolism upon N limitation, provides more carbon flux to acetyl-CoA and NADPH for fatty acid biosynthesis.

**Electronic supplementary material:**

The online version of this article (doi:10.1186/s12934-016-0428-4) contains supplementary material, which is available to authorized users.

## Background

Oleaginous microorganisms include fungi, yeasts, microalgae and bacteria that can accumulate oil to more than 20 % of their cell dry weight (CDW) [1]. The metabolism of lipid accumulation in oleaginous microorganisms has been extensively studied as microbial oils can be used as commercial sources of several nutritionally-important polyunsaturated fatty acids (PUFAs) and as potential sources of biofuels [2, 3]. Among oleaginous filamentous fungi, *Mucor circinelloides* has been considered as an important model organism for lipid accumulation studies due to its ability to produce an oil rich in γ-linolenic acid (GLA, 18:3; n-6), that may have beneficial effects for the treatment of premenstrual tension, atopic dermatitis and some other diseases [4] and also due to the availability of genome data and genetic tools.

Lipid accumulation in oleaginous microorganisms is triggered by a nutrient imbalance in the culture medium. When cells run out of a key nutrient, usually nitrogen (N), excess carbon substrate continues to be assimilated by the cells and converted into storage lipids [3]. The biochemistry of lipid accumulation in oleaginous microorganisms triggered by N deficiency has been widely investigated. Previous data indicated that the activity of isocitrate dehydrogenase decreases rapidly and even ceases completely under N-deficient conditions as the diminishing concentration of its allosteric regulator AMP, and then results in a shift in carbon flux through the citric acid cycle and into lipid biosynthesis [5, 6]. In addition, ATP: citrate lyase (ACL), which generates acetyl-CoA as the precursor of fatty acids via the cleavage of citric acid, is an essential enzyme for fatty acid biosynthesis and possibly catalyzes the rate-limiting reaction for fatty acid biosynthesis in some oleaginous organism [7–9].

Beside acetyl-CoA, the provision of reducing power in the form of NADPH is another critical process for fatty acid biosynthesis. The key roles of malic enzyme and the pentose phosphate pathway (glucose-6-phosphate dehydrogenase coupled with 6-phosphogluconate dehydrogenase) to supply NADPH for fatty acid synthesis during lipid accumulation have been proposed [2, 10–17]. However, lipid accumulation is a complicated process involving many metabolic pathways and thus it is impossible to achieve maximal lipid production simply by regulating just one or two genes. An investigation of lipid metabolism at systematic level is therefore required to gain insights into the molecular mechanism of lipid accumulation.

Cellular responses to N deficiency are the subtle behaviors of living organisms. Although there are alternative approaches to understand the molecular mechanisms of cellular response under N deficiency, microbial proteomics has become a powerful tool to investigate the complex cellular processes. In addition, it can also determine new functions of gene products as it represents not only the gene product, but also translational rate and post-translational modifications. Identification of proteins that are up/down-regulated under N deficiency is important in studying the mechanisms of lipid accumulation. Accordingly, comparative proteomics have been explored to gain insights into the lipid metabolism under N deficiency in microalgae and yeast [18–21].

*M. circinelloides* was the first microorganism to be used commercially to produce an oil for human consumption—an oil rich in GLA [4]. However, the process began in 1985 but lasted only 6 years as the commercial strain only produced 25 % lipid of its cell biomass and at that time high GLA-producing plant species came on to the market [3, 4]. In this study, we performed a comparative proteomic study on the oleaginous fungus *M. circinelloides* WJ11, which is the highest lipid-producing strain (up to 36 % lipid, w/w) of this species as far and its lipid content is much higher than that commercial strain [6]. *M. circinelloides* WJ11 could be the potential strain to produce the commercial oil rich in GLA and its mechanism of lipid accumulation at proteomic level will provide a foundation of restarting the commercial production of GLA by microorganism. We compared proteomes from three growth stages (the balanced growth stage, the fast lipid accumulation stage and the slow lipid accumulation stage) to provide new insights into the mechanism of lipid metabolism in this fungus. To the best of our knowledge, this is the first proteomic study on the lipid overproduction process of the oleaginous filamentous fungus, *M. circinelloides* WJ11, under N deficiency.

## Results and discussion

### Cell growth and lipid accumulation in *M. circinelloides* WJ11

**Fig. 1:**
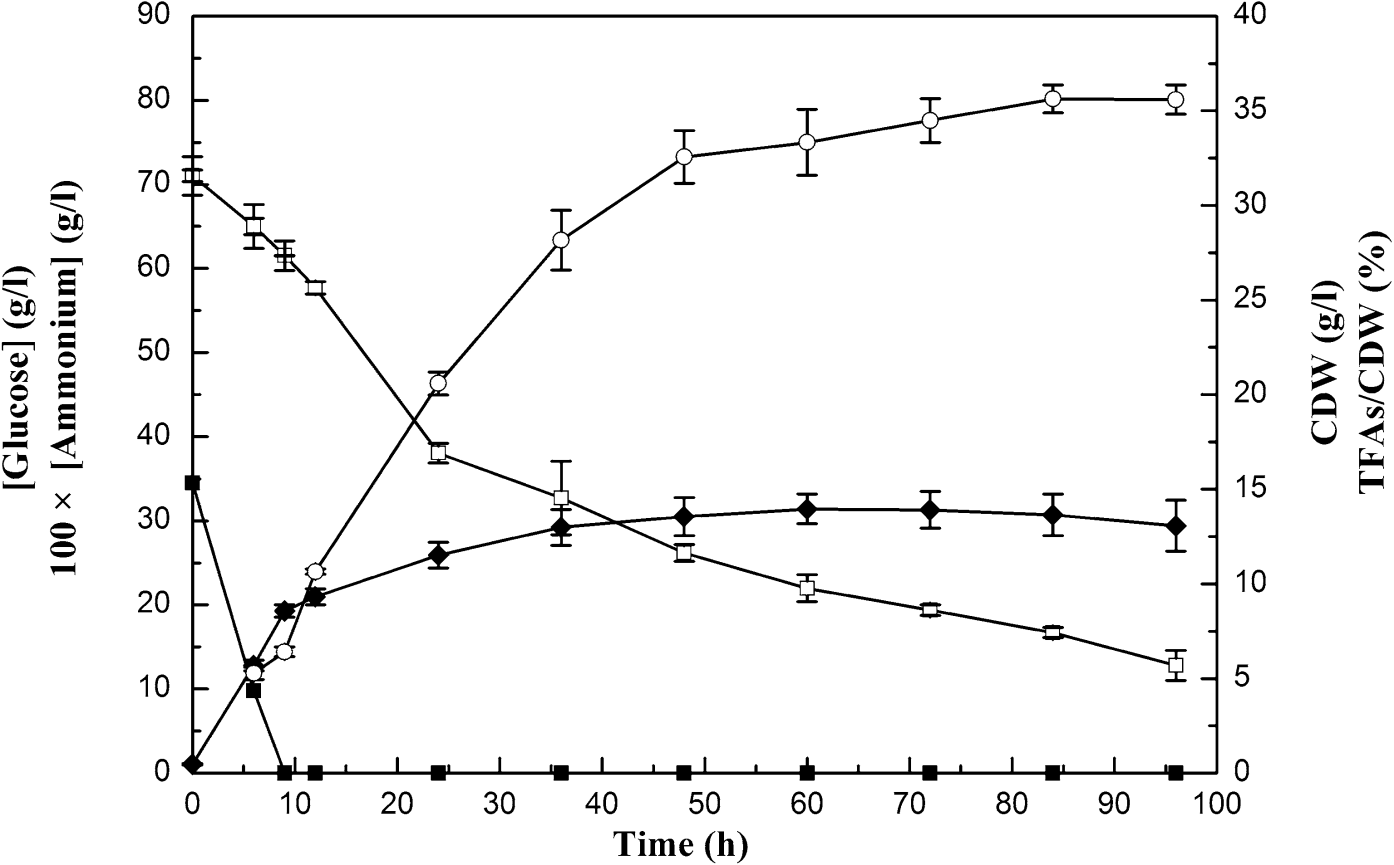
Cell growth and lipid accumulation of *M. circinelloides* WJ11. Ammonium and glucose concentration in the growth media, cell dry weight (CDW) and total fatty acids (TFAs) content (w/w, CDW) of strain WJ11 in the modified K & R medium in 2L fermenter. *Open square* glucose concentration; *filled square* ammonium concentration, *filled diamond* CDW, *open circle* TFAs/CDW. Values were measured as mean of three biological replicates. *Error bars* represent the standard error of the mean

In most oleaginous microorganisms, the amount of lipid increases under N starvation and lipid accumulation is often investigated by comparing the N rich phase to N deficiency phase in the entire bioprocess [10, 18, 22]. The concentrations of ammonium and glucose in culture medium, cell dry weight (CDW), and lipid accumulation of *M. circinelloides* WJ11 during growth are shown in Fig. 1. Ammonium was used up at approx. 9 h and glucose remained in excess during the entire bioprocess. CDW initially increased rapidly up to 9 h of growth, and then slowed down after nitrogen exhaustion. Immediately after nitrogen depletion from the growth medium, the fungus started to accumulate lipids; from 9 to 48 h, the total fatty acids (TFAs) content increased rapidly and then slowed. The maximal TFAs content in *M. circinelloides* WJ11 was 36 % CDW; this was considerably greater than that in other strains of the fungus: CBS 277.49 (10–15 %, w/w) and CBS 108.16 (20–25 %, w/w) [10, 23, 24]. In this study, the cells of *M. circinelloides* WJ11 were collected at 6 h (N rich and balanced growth stage), 24 h (after N depletion and fast lipid accumulation stage) and 60 h (after N depletion and slow lipid accumulation stage) for further research.

Furthermore, we also calculated the average rate of lipid biosynthesis in *M. circinelloides* WJ11 during the bioprocess (Table 1). The result showed the rate of lipid production goes up after N exhaustion at 9 h (the rate from 12 to 24 h is higher than from 6 to 9 h), and decreases after 36 h.

**Table 1:** Average lipid biosynthesis rate in *M. circinellodies* WJ11 during the bioprocess

Time	Average lipid biosynthesis rate^a^ (mg g^−1^ h^−1^)
From 6 to 9 h	12.4
From 9 to 12 h	18
From 12 to 24 h	13.1
From 24 to 36 h	11.5
From 36 to 48 h	6.8
From 48 to 60 h	2.2
From 60 to 72 h	1.3
From 72 to 84 h	0.6
From 84 to 96 h	0

^a^Average lipid biosynthesis rate was calculated by the synthesized lipid per gram lipid-free cell dry weight per hour

### Proteome analysis of *M. circinelloides* during lipid accumulation upon N deficiency

**Fig. 2:**
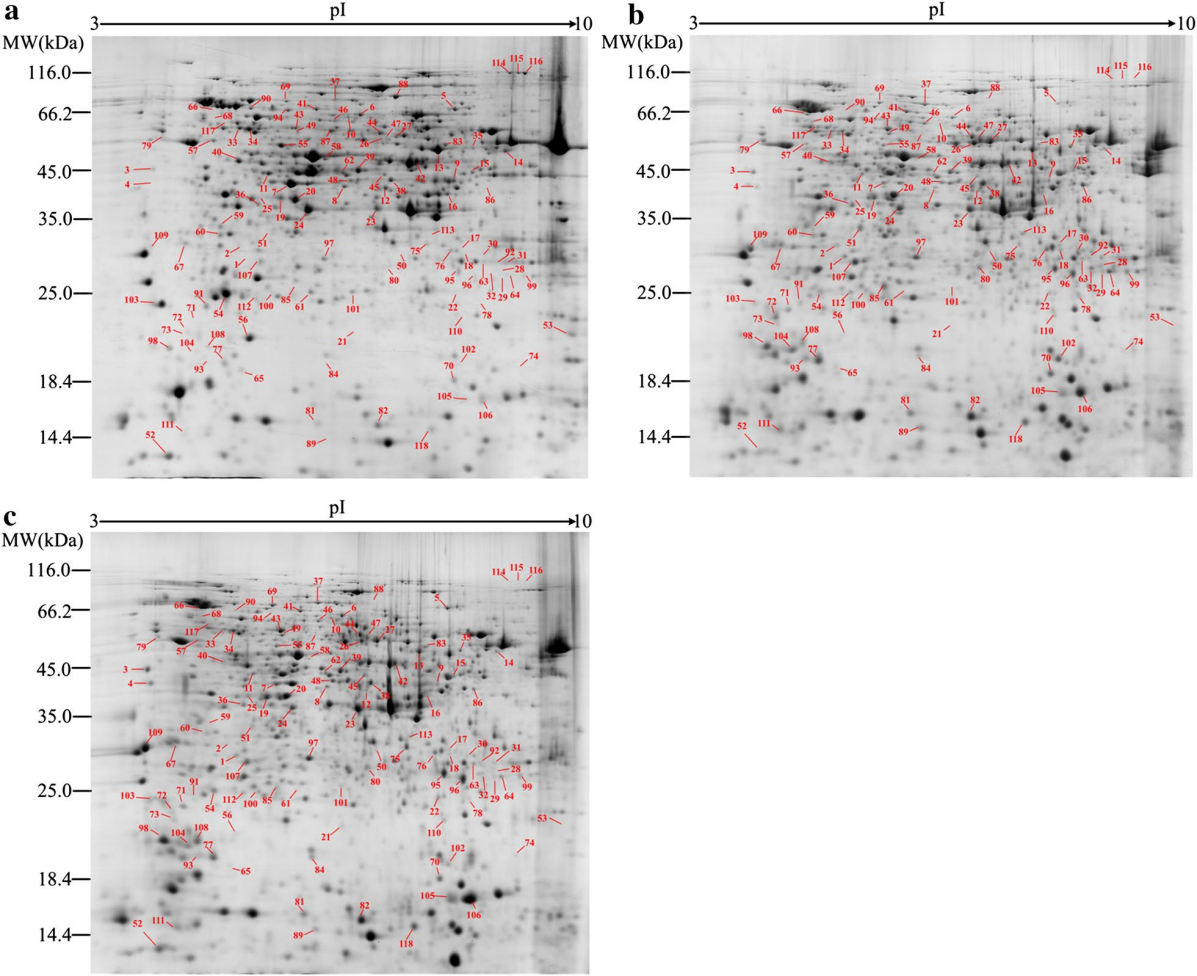
Comparative analysis of the intracellular proteome in *M. circinelloides* WJ11. **a** 6 h, **b** 24 h, **c** 60 h. The differentially expressed proteins are marked by numbers

To investigate the differentially expressed proteins of *M. circinelloides* WJ11 during lipid accumulation triggered by N depletion, proteomic analysis was applied by 2-DE at 6, 24 and 60 h, which, respectively, represent three growth stages: balanced growth stage (3 h before N depletion); the fast lipid accumulation phase (15 h after N depletion); and the slow lipid accumulation phase (51 h after N depletion). The representative gels of protein spots from the different stages are shown in Fig. 2. More than 800 spots were detected in each gel and 118 of these spots showed significant changes (>1.5-fold or <0.67-fold) under N deficiency. The differential protein spots were excised from the gels for MALDI-TOF/TOF MS analyses and these identified proteins were shown in Table 2.

**Table 2:** Identification of differentially expressed proteins under N deficiency in *M. circinellodies* WJ11 by MALDI-TOF/TOF analysis

Spot	Accession	Protein name	Theoretical MW (Da)/pI	MASCOT score	No. matched	Coverage (%)	Fold change 24 h/6 h	Fold change 60 h/6 h
*Nitrogen metabolism*								
1	gi|511001426	Glutamine synthetase	39,973/5.61	73	2 (0)	6	14.49	5.58
2	gi|511003862	Glutamine synthetase	40,412/5.61	102	2 (2)	11	15.04	2.79
3	gi|511008284	Saccharopepsin	44,921/4.81	243	5 (2)	17	37.86	60.50
4	gi|511001898	Saccharopepsin	44,736/4.91	129	3 (0)	8	36.84	91.88
*Amino acid metabolism*								
5	gi|511002358	Acetolactate synthase	73,772/8.13	297	5 (1)	10	0.02	1.03
6	gi|511004294	Dihydroxy-acid dehydratase	64,148/6.27	432	8 (4)	11	0.55	0.82
7	gi|511002359	Ketol-acid reductoisomerase	44,298/7.72	562	9 (3)	27	0.10	0.17
8	gi|511004962	Branched-chain amino acid aminotransferase	43,753/6.62	474	6 (3)	19	0.38	0.59
9	gi|511010054	Acetylornithine aminotransferase	49,342/8.32	123	4 (1)	15	0.75	0.37
10	gi|511006376	Asparagine synthetase	64,754/5.81	88	3 (0)	8	0.15	0.80
11	gi|511005296	Saccharopine dehydrogenase	41,899/5.36	471	12 (3)	35	0.38	0.29
12	gi|511002126	Homoisocitrate dehydrogenase	41,047/6.28	102	3 (0)	10	0.29	0.15
13	gi|511009910	Serine hydroxymethyltransferase 2	51,589/6.53	193	8 (2)	24	0.39	0.31
14	gi|511001743	Serine hydroxymethyltransferase, mitochondrial	55,707/8.97	109	4 (1)	11	0.30	0.22
15	gi|511000252	Glycine cleavage system T protein	43,694/8.77	82	4 (0)	11	0.18	0.47
16	gi|511004260	3-Deoxy-7-phosphoheptulonate synthase	38,738/6.46	239	4 (1)	9	0.20	0.10
17	gi|511010625	*S*-Adenosylmethionine synthase	42,413/5.67	70	1 (1)	3	5.39	3.55
18	gi|511010625	*S*-Adenosylmethionine synthase	42,413/5.67	106	1 (1)	3	3.22	1.41
*Carbon metabolism*								
19	gi|511002640	Fructose-bisphosphate aldolase	39,791/5.36	145	2 (2)	11	1.64	2.99
20	gi|511002640	Fructose-bisphosphate aldolase	39,791/5.36	236	3 (1)	11	1.55	1.51
21	gi|511008895	Glyceraldehyde-3-phosphate dehydrogenase 1	35,588/6.09	88	3 (0)	10	2.77	7.56
22	gi|511008895	Glyceraldehyde-3-phosphate dehydrogenase 1	35,588/6.09	106	2 (0)	5	8.24	13.63
23	gi|511008895	Glyceraldehyde-3-phosphate dehydrogenase 1	35,588/6.09	394	5 (3)	15	2.50	3.50
24	gi|511007306	Glyceraldehyde-3-phosphate dehydrogenase 2	36,013/5.54	550	10 (5)	35	2.91	1.69
25	gi|511004614	Enolase	47,164/5.56	385	5 (2)	10	1.53	7.99
26	gi|511006733	Pyruvate kinase	59,079/6.19	464	8 (3)	18	7.59	6.44
27	gi|511006733	Pyruvate kinase	59,079/6.19	109	4 (0)	3	4.23	3.97
28	gi|511004430	Pyruvate dehydrogenase complex	52,323/6.11	170	2 (1)	5	17.82	1.36
29	gi|511004430	Pyruvate dehydrogenase complex	53,161/5.76	123	2 (1)	5	21.82	4.58
30	gi|511005313	Pyruvate dehydrogenase E1 component subunit alpha	43,770/8.31	246	6 (2)	11	6.89	1.35
31	gi|511002644	Pyruvate dehydrogenase E2 component	53,161/5.76	183	4 (1)	8	1.93	1.51
32	gi|511002644	Pyruvate dehydrogenase E2 component	53,161/5.76	288	4 (2)	8	50.48	21.50
33	gi|511003312	2,3-Bisphosphoglycerate-independent phosphoglycerate mutase	56,737/5.23	332	8 (3)	16	0.48	0.41
34	gi|511003312	2,3-Bisphosphoglycerate-independent phosphoglycerate mutase	56,737/5.23	118	3 (0)	5	0.46	0.62
35	gi|511010411	Glucose-6-phosphate dehydrogenase	58,358/6.72	96	3 (0)	12	1.62	1.51
36	gi|511005381	Transaldolase	35,798/5.45	137	3 (0)	9	2.64	0.71
37	gi|511001250	Transketolase	74,273/5.71	108	4 (1)	8	2.64	2.56
38	gi|511005018	NAD^+^:isocitrate dehydrogenase, mitochondrial	41,006/7.02	735	11 (7)	34	0.53	0.62
39	gi|511005320	NADP^+^:isocitrate dehydrogenase, mitochondrial	47,644/5.97	164	4 (1)	11	0.31	0.57
40	gi|511002271	Succinyl-CoA ligase, mitochondrial	47,555/5.89	138	4 (1)	12	0.55	0.39
41	gi|511009244	Succinate dehydrogenase	71,166/6.15	112	3 (1)	7	0.71	0.55
42	gi|511006629	Fumarate hydratase, mitochondrial	53,753/6.65	410	4 (4)	11	0.55	0.39
43	gi|511003684	Aldehyde dehydrogenase	54,639/5.51	368	10 (4)	20	1.52	1.95
44	gi|511001067	Aldehyde dehydrogenase	53,854/5.57	284	5 (2)	14	1.64	1.72
45	gi|511008662	Acetyl-CoA C-acetyltransferase	41,434/5.65	507	8 (4)	23	0.39	0.43
46	gi|511005081	Phosphoglucomutase	61,303/5.61	151	4 (1)	11	3.50	4.55
47	gi|511006219	UTP-glucose-1-phosphate uridylyltransferase	56,716/6.15	655	10 (3)	23	3.21	4.01
48	gi|511006325	UDP-glucose 4-epimerase	39,235/5.76	138	3 (0)	12	1.07	1.55
49	gi|511009038	Galactokinase	48,847/6.00	209	5 (0)	12	2.17	2.26
50	gi|511010174	*S*-Formylglutathione hydrolase	32,160/6.11	61	2 (0)	3	2.27	2.68
51	gi|758354396	Thiazole biosynthetic enzyme	33,882/5.28	345	3 (1)	18	20.96	14.71
*Protein metabolism*								
52	gi|511000792	40S ribosomal protein S21	9718/4.93	287	3 (2)	66	0.34	0.35
53	gi|511011908	40S ribosomal protein S7	21,057/9.70	71	1 (1)	12	0.07	0.12
54	gi|511008091	Eukaryotic translation initiation factor 5A	17,694/5.08	302	3 (3)	19	0.13	0.68
55	gi|511011759	Elongation factor Tu	51,841/6.26	294	5 (2)	13	0.17	0.09
56	gi|511006438	Nascent polypeptide-associated complex subunit beta	17,501/5.34	362	5 (3)	48	0.02	0.06
57	gi|511003551	26S protease regulatory subunit 6A	48,455/4.96	346	9 (2)	18	0.29	0.33
58	gi|511009637	26S protease regulatory subunit 6B	46,545/5.72	228	4 (2)	15	0.42	0.38
59	gi|511010098	20S proteasome subunit Alpha 6	31,144/5.06	254	4 (2)	15	0.01	0.02
*Nucleic acid metabolism*								
60	gi|511005974	Phosphoribosylaminoimidazole-succinocarboxamide synthase	33,792/5.10	231	3 (2)	11	0.49	0.60
61	gi|511011778	Adenylyl-sulfate kinase	22,883/5.75	387	8 (2)	32	0.20	0.12
62	gi|511006145	Dihydroorotase, homodimeric type	39,652/5.83	233	5 (0)	13	0.43	0.45
*Energy metabolism*								
63	gi|511006698	Adenylate kinase 1	28,268/7.01	624	10 (6)	51	1.61	2.53
64	gi|511006698	Adenylate kinase 1	28,268/7.01	571	8 (5)	29	37.78	20.29
65	gi|511003961	V-type H+-transporting ATPase subunit I	95,461/5.05	193	3 (1)	5	1.61	1.51
66	gi|511007631	V-type proton ATPase catalytic subunit A	70,756/5.07	361	6 (3)	15	3.23	1.72
*Signal transduction*								
67	gi|511007036	14-3-3 family protein epsilon	29,228/4.84	175	5 (1)	24	1.75	2.01
*Molecular chaperone*								
68	gi|511004981	Heat shock 70 kDa protein 1/8	67,760/5.10	280	3 (2)	7	4.23	3.64
69	gi|511004335	Hsp70-like protein	71,389/5.79	202	4 (1)	7	2.38	2.99
70	gi|511003138	Peptidyl-prolyl cis–trans isomerase cyp5	19,078/6.92	477	8 (5)	47	1.72	1.64
*Redox homeostasis*								
71	gi|511008071	Peroxiredoxin	22,926/4.94	254	3 (2)	25	20.46	2.95
72	gi|511008071	Peroxiredoxin	22,926/4.94	114	2 (1)	12	15.30	13.16
73	gi|511008071	Peroxiredoxin	22,926/4.94	173	3 (1)	14	16.70	17.01
74	gi|503389911	Glutathione peroxidase	20,881/6.40	61	1 (0)	5	1.58	3.42
75	gi|511008344	Oxidoreductase	30,743/6.25	432	5 (2)	15	1.55	3.44
76	gi|511009098	Oxidoreductase	28,164/6.54	310	6 (2)	28	1.84	1.66
77	gi|511009792	Ferritin heavy chain	20,301/5.23	268	5 (3)	39	2.30	2.08
*Hypothetical protein*								
78	gi|511011920	Hypothetical protein HMPREF1544_00199	91,261/6.42	211	5 (1)	5	23.83	8.50
79	gi|511010665	Hypothetical protein HMPREF1544_01177	55,006/4.72	85	2 (1)	6	4.84	2.65
80	gi|511010454	Hypothetical protein HMPREF1544_01386	28,881/6.42	101	2 (0)	6	41.34	58.10
81	gi|511009952	Hypothetical protein HMPREF1544_01888	14,839/5.89	125	1 (1)	10	4.23	2.59
82	gi|511009952	Hypothetical protein HMPREF1544_01888	14,839/5.89	293	3 (2)	32	3.18	1.74
83	gi|511009799	Hypothetical protein HMPREF1544_02101	47,569/7.79	191	3 (2)	10	0.35	0.43
84	gi|511009692	Hypothetical protein HMPREF1544_02181	22,351/5.33	175	3 (2)	13	23.25	37.32
85	gi|511009605	Hypothetical protein HMPREF1544_02260	26,248/5.37	175	3 (1)	12	9.70	3.04
86	gi|511008946	Hypothetical protein HMPREF1544_03035	42,241/7.22	630	8 (4)	26	34.49	15.14
87	gi|511008765	Hypothetical protein HMPREF1544_03136	53,353/5.77	422	5 (4)	15	0.06	0.07
88	gi|511008618	Hypothetical protein HMPREF1544_03262	68,758/6.29	378	7 (3)	12	0.01	0.01
89	gi|511008350	Hypothetical protein HMPREF1544_03549	43,856/7.82	229	4 (2)	9	13.89	17.99
90	gi|511008286	Hypothetical protein HMPREF1544_03639	48,153/5.50	235	5 (2)	10	0.11	0.25
91	gi|511008150	Hypothetical protein HMPREF1544_03790	20,026/5.04	307	4 (2)	22	0.16	0.34
92	gi|511008020	Hypothetical protein HMPREF1544_03919	29,429/6.97	425	6 (4)	37	0.20	0.19
93	gi|511007940	Hypothetical protein HMPREF1544_03956	29,843/5.14	123	2 (1)	10	25.34	20.07
94	gi|511007175	Hypothetical protein HMPREF1544_04707	63,905/5.35	106	2 (0)	4	0.09	0.09
95	gi|511006887	Hypothetical protein HMPREF1544_05024	55,228/6.28	275	3 (2)	9	2.16	5.78
96	gi|511006887	Hypothetical protein HMPREF1544_05024	55,228/6.28	309	5 (2)	10	5.47	5.35
97	gi|511006017	Hypothetical protein HMPREF1544_05863	30,010/5.58	423	4 (4)	23	2.45	2.18
98	gi|511004978	Hypothetical protein HMPREF1544_06871	18,967/4.96	249	3 (2)	13	5.27	17.90
99	gi|511004902	Hypothetical protein HMPREF1544_06986	28,947/9.05	103	1 (1)	6	3.61	2.41
100	gi|511004522	Hypothetical protein HMPREF1544_07306	26,114/5.56	252	4 (2)	21	7.99	3.58
101	gi|511004398	Hypothetical protein HMPREF1544_07438	21,313/5.70	199	3 (1)	13	0.29	0.18
102	gi|511004099	Hypothetical protein HMPREF1544_07752	21,249/5.14	294	6 (2)	31	24.54	3.86
103	gi|511003967	Hypothetical protein HMPREF1544_07893	16,356/4.59	152	3 (1)	30	0.04	0.20
104	gi|511003188	Hypothetical protein HMPREF1544_08616	30,249/5.39	224	4 (1)	11	28.24	26.23
105	gi|511003039	Hypothetical protein HMPREF1544_08758	19,098/8.39	392	4 (3)	25	4.40	2.25
106	gi|511003039	Hypothetical protein HMPREF1544_08758	19,098/8.39	347	8 (2)	70	8.61	9.91
107	gi|511002615	Hypothetical protein HMPREF1544_09175	26,203/5.36	170	4 (1)	22	6.71	3.31
108	gi|511002606	Hypothetical protein HMPREF1544_09235	58,903/4.89	98	1 (1)	5	23.78	30.46
109	gi|511002606	Hypothetical protein HMPREF1544_09235	58,903/4.89	81	2 (0)	5	5.99	20.72
110	gi|511001781	Hypothetical protein HMPREF1544_10038	43,811/6.02	96	1 (1)	4	8.25	14.78
111	gi|511001506	Hypothetical protein HMPREF1544_10287	14,404/4.95	156	3 (0)	26	24.12	22.30
112	gi|511001144	Hypothetical protein HMPREF1544_10622	24,389/5.25	162	3 (1)	15	5.08	4.08
113	gi|511001065	Hypothetical protein HMPREF1544_10686	38,562/6.58	161	5 (0)	20	26.39	17.31
114	gi|511000884	Hypothetical protein HMPREF1544_10852	100,320/8.41	161	3 (1)	5	0.04	0.00
115	gi|511000884	Hypothetical protein HMPREF1544_10852	100,320/8.41	484	6 (4)	10	0.04	0.01
116	gi|511000884	Hypothetical protein HMPREF1544_10852	10,0320/8.41	210	4 (1)	7	0.01	0.02
117	gi|511000397	Hypothetical protein HMPREF1544_11327	66,634/5.41	127	3 (0)	8	24.47	16.26
118	gi|511000331	Hypothetical protein HMPREF1544_11393	14,081/6.75	138	3 (1)	38	2.61	3.05

### Nitrogen and amino acid metabolism

Glutamine synthase (GS) is the key enzyme involved in ammonia assimilation in both plants and microorganisms [25, 26]. Previous proteomic analysis has indicated that its expression is induced immediately following N deprivation in both *Phaeodactylum tricornutum* and *Rhodosporidium toruloides* [18, 21]. Indeed, our result showed its expression (spots 1 and 2) in *M. circinelloides* was up-regulated upon N depletion. In oleaginous yeasts, upon N deficiency and at the beginning of lipid accumulation, AMP is deaminated to release ammonium for further cell use. Thus, the substrate NH_4_^+^ for GS might be from the degradation of AMP or other N compounds. Saccharopepsin (spots 3 and 4), which links to vacuolar protein degradation, was also induced upon N depletion in this study. The elevated expression of this protein would enhance degradation of proteins suggesting that, immediately after N exhaustion, the cells initiate the turnover and recycling of intracellular components-especially proteins that are no longer needed for anabolic reactions [12].

Most of the proteins associated with amino acid metabolism showed decreased expression level after N depletion. Acetolactate synthase (spot 5), dihydroxy-acid dehydratase (spot 6) and ketol-acid reductoisomerase (spot 7) catalyze the synthesis of the branched-chain amino acids (BCAA, e.g., valine, leucine, and isoleucine) [27–29], and branched-chain amino acid aminotransferase (spot 8) participates in the degradation BCAA [30]. These proteins related to BCAA metabolism were all down-regulated upon N depletion. Acetylornithine aminotransferase (spot 9) and asparagine synthetase (spot 10) are involved in the asparagine biosynthesis [31]. Saccharopine dehydrogenase (spot 11) and homoisocitrate dehydrogenase (spot 12) participate in lysine biosynthesis [32, 33]. Serine hydroxymethyltransferase (spots 13 and 15) take part in serine degradation and glycine cleavage system T protein (spot 15) is involved in glycine degradation [34]. 3-deoxy-7-phosphoheptulonate synthase (spot 16) is responsible for the biosynthesis of phenylalanine, tyrosine, and tryptophan. These above proteins related to metabolism of amino acids were also down-regulated upon N depletion. Decreased expression of the proteins associated with amino acid metabolism indicated that amino acid biosynthesis was at least partially inhibited due to the absence of nitrogen. In these proteins related to amino acid metabolism, one exception to the overall decreased expression was *S*-adenosylmethionine synthase (SAS, spots 17 and 18) which participates in the biosynthesis of *S*-adenosylmethionine, which is a precursor of glutathione and other methylated cell components [35].

N deprivation can result in a metabolic imbalance of reactive oxygen species (ROS) and an excess of oxygen radicals [36], while glutathione can quench free radicals and then improve the resistance of the cells to this stress [37]. Therefore, the increased expression of SAS could play a role in providing glutathione to improve stress resistance arising during N deficiency.

### Carbon metabolism

**Fig. 3:**
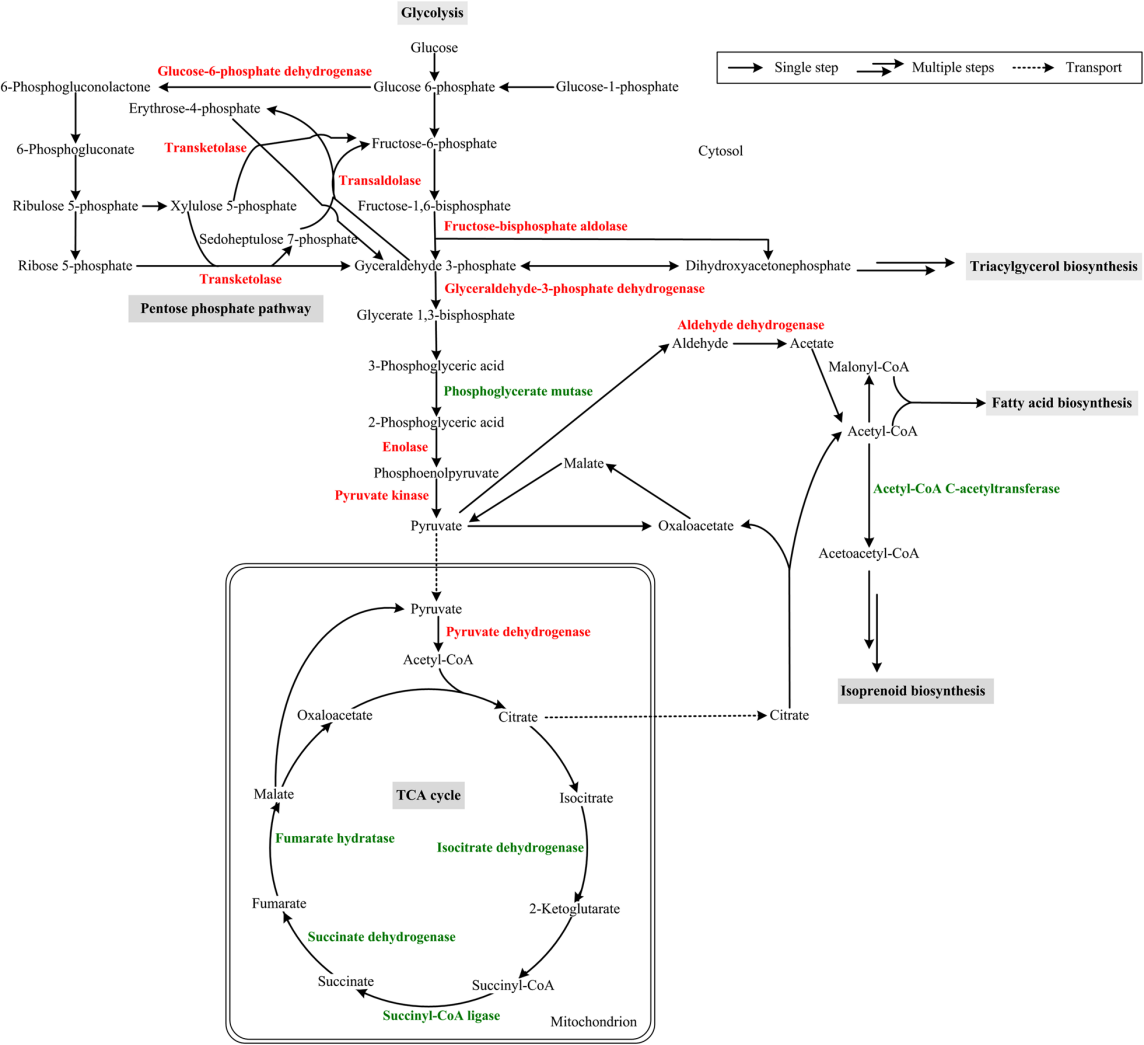
Networks of carbon metabolism related to lipid accumulation in *M. circinelloides* WJ11. The proteins marked as *red* were up-regulated and which marked as *green* were down-regulated under N deficiency

Carbon metabolism and flux are critical to lipid accumulation. *M. circinelloides* can grow well and accumulate abundant lipids using glucose as the single carbon source [6, 10, 24, 38]. The differential expression of proteins involved in central carbon metabolism pathway is shown in Fig. 3.

The glycolytic pathway provides pyruvate, a key precursor for acetyl-CoA, which is the substrate for fatty acid biosynthesis. Fructose-bisphosphate aldolase (FBA, spots 19 and 20), an essential enzyme involved in glycolysis that catalyzes a reversible cleavage reaction of fructose 1,6-bisphosphate into glyceraldehyde 3-phosphate (G3P) and dihydroxyacetone phosphate (DHAP) [39], was up-regulated under N deficiency. The increased FBA activity could stimulate glycolysis and triacylglycerol biosynthesis. Glyceraldehyde-3-phosphate dehydrogenase (spots 21, 22, 23 and 24), enolase (spots 25) and pyruvate kinase (spots 26 and 27), which are key enzymes in the glycolytic pathway, were also up-regulated under N deficiency. Up-regulation of these proteins may also lead to the increased production of pyruvate. In addition, the expression of pyruvate dehydrogenase (PDH, spots 28, 29, 30, 31 and 32) was increased and thus lead to increased conversion of pyruvate into acetyl-CoA in the mitochondrion. Taken together, under N deficiency, the expression of many proteins involved in glycolytic pathway was increased, which would then provide more acetyl-CoA in the mitochondrion.

Phosphoglycerate mutase (PGM, spots 33 and 34) is also involved in glycolysis, catalyzing the interconversion of 3-phosphoglycerate (3PGA) to 2-phosphoglycerate (2PGA). In this study, PGM was down-regulated when the fungus was grown under N deficient condition, as FBA was up-regulated, the expression changes of these proteins could lead to accumulation of some intermediate between fructose-1,6-bisphosphate and 3-phosphoglyceric acid and this could be glycerol.

The pentose phosphate pathway (PPP) generates NADPH, which is an important source besides malic enzyme (ME) for providing reducing power for fatty acid biosynthesis [2]. In this study, glucose-6-phosphate dehydrogenase (G6PDH, spot 35), which is a key enzyme in the PPP that generates NADPH, was up-regulated under N deficiency. This is in accordance with our previous report showing that lipid accumulation in *M. circinelloides* is accompanied by increased G6PDH activity after N depletion from the medium [6]. Furthermore, transaldolase (spot 36) and transketolase (spot 37), as part of the PPP, were both up-regulated. These results indicated that PPP was more active during the lipid accumulation phase after N depletion, and this would provide more NADPH for fatty acid biosynthesis. For ME, the critical enzyme plays a key role in supplying NADPH for fatty acid synthesis and desaturation in *M. circinelloides* [10, 38, 40, 41], we did not find any change in its expression at proteomic level upon N deficiency, which indicates the ME gene is being expressed all the time, irrespective of the status of the cells, and regulation of ME maybe complicated.

Oleaginous yeasts deaminate AMP to release ammonium and IMP upon nitrogen limitation [3]. NAD^+^:isocitrate dehydrogenase (NAD^+^:ICDH) requires AMP for activity. With ICDH activity being curtailed by the lack of AMP, isocitrate accumulates and equilibrates back to citrate which is then transported out of the mitochondrion into the cytosol, and thus provides the increased carbon flux to acetyl-CoA for fatty acid synthesis [5]. In this study, the NAD^+^:ICDH (spot 38) was down-regulated upon N deficiency which would increase citrate accumulation and then lead to greater carbon flux to acetyl-CoA for fatty acid synthesis. Mitochondrial NADP^+^:ICDH (spot 39) was also down-regulated, and this regulation was similar to NAD^+^:ICDH. In addition, the expression of some other proteins (e.g., succinyl-CoA ligase, spot 40; succinate dehydrogenase, spot 41; fumarate hydratase, spot 42) involved in TCA cycle were all decreased, and this further suggests that the TCA cycle is retarded upon N deficiency which will therefore lead to a greater carbon flux to lipids biosynthesis. These results are well in accordance with our previous studies of lipid accumulation in *M. circinelloides* and the multi-omic analysis of lipid accumulation in *Rhodosporidium toruloides* by Zhu et al. [6, 12].

Aldehyde dehydrogenase (ALDH, spots 43 and 44), which converts acetaldehyde into acetate, was up-regulated upon N deficiency. Although in oleaginous fungi and higher eukaryotes, the major route of acetyl-CoA production is by the cleavage of citrate by ATP:citrate lyase (ACL) [7], it can also be produced by cytoplasmic acetyl-CoA synthase (ACS) from acetate when acetate is being produced by the degradation of proteins and amino acids that are surplus to the requirements of the cell. The level of acetyl-CoA C-acetyltransferase (ACAT, spot 187), which is the branch point enzyme for acetyl-CoA to synthesize isoprenoids, was decreased under N deficiency which indicates that the acetyl-CoA flux is being preferentially switched into the synthesis of fatty acids but not into isoprenoids.

Galactokinase (spot 49) catabolizes β-D-galactose to glucose 1-phosphate. Phosphoglucomutase (spot 46) facilitates the interconversion of glucose 1-phosphate and glucose 6-phosphate. UTP-glucose-1-phosphate uridylyltransferase (spot 47) synthesizes UDP-glucose from glucose-1-phosphate. UDP-glucose 4-epimerase (spot 48) catalyzes the reversible conversion of UDP-galactose to UDP-glucose. These proteins are involved in galactose and glucose conversion into polysaccharides and were all up-regulated upon N deficiency, suggesting that glucose utilization of the fungus might be sequentially proceeded during lipid accumulation triggered by N depletion.

*S*-Formylglutathione hydrolase (spot 50) can produce glutathione from hydrolysis of *S*-formylglutathione and its expression was up-regulated upon N deficiency. N deprivation can result in an excess of oxygen radicals and glutathione can quench free radicals [42]. Therefore the up-regulated protein could be a stress response to N deficiency. Thiazole biosynthetic enzyme (TBE, spot 51) associated with thiamine metabolism was also up-regulated upon N depletion. It plays additional roles in adaptation to various stress conditions and in DNA damage tolerance [43, 44]. Thus the increased expression of TBE in the fungus is likely the cellular adaptation to N depletion.

### Other metabolism

As de novo protein biosynthesis is no longer occurring after N deficiency, then the cell must conserve its key proteins for as long as possible. Thus, the expression of proteins (spots 52–59) related to protein metabolism were diminished in the fungus under N deficient conditions. In addition, the expression of proteins involved in nucleic acid metabolism (spots 60, 61 and 62) were also decreased and this further indicates that cell reproduction and growth are decreased upon N depletion [19].

The activity of adenylate kinase has been found to be stimulated after N-exhaustion in *M. circinelloides* [45] and we found that, in accordance with this, the expression of adenylate kinase (spots 63 and 64) was increased upon N deficiency. Furthermore, ATPase (spots 65 and 66) was also up-regulated. The metabolic pattern of *M. circinelloides* is therefore readjusted when N becomes exhausted in the medium. In *M. circinelloides*, the concentrations of ATP, ADP and AMP decrease at the time of N-exhaustion so that energy, in the form of ATP, is now produced at a lower level [20, 45]. Therefore the increased expression of adenylate kinase and ATPase might play a role in helping to maintain energy production.

14-3-3 Family protein epsilon (spot 67) can restrain cell apoptosis and promote cell survival under stress condition [46] and the increased expression in N limitation could be the cellular response to N deficiency. Heat shock proteins (HSPs) are a group of functionally related proteins responsible for protein folding and unfolding. High-level expression of HSPs can be triggered by exposure to different environmental stress conditions, including exposure of the cell to nitrogen deficiency [47]. We identified similar trends in N-deprived cells, including two HSPs (spots 68 and 69), which were significantly up-regulated. Peptidyl-prolyl *cis*–*trans* isomerase plays roles in protein folding and transport, RNA splicing and the regulation of multi-protein complexes in cells [48]. In this study, expression of this protein (spot 70) was up-regulated in N deprivation, and this might also be part of the cellular adaption to stress conditions engendered by N deficiency.

Excessive generation of ROS or oxidative stress is an integral part of many stress situations, including N limitation [36]. Peroxiredoxin (Prx) is a ubiquitous family of antioxidant enzymes and glutathione peroxidase (GPx) has the biological role in protecting organism from oxidative damage. Indeed, the Prx (spots 71, 72 and 73) and GPx (spot 74) were up-regulated upon N deficiency. The increased expression of an oxidoreductase (spots 75 and 76) that catalyzes the transfer of electrons from reductant to oxidant is also a response to oxidative stress upon N limitation. Ferritin heavy chain (spot 77), a ubiquitous and highly conserved protein, which plays a major role in iron homeostasis, was also up-regulated upon N deficiency. Many haem-proteins would be degraded as being surplus to the requirements of the cell during N deprivation. The iron being released from these proteins and other non-haem iron proteins will therefore be scavenged by the cell and stored intracellularly in ferritin. This may lead to the increased expression of ferritin heavy chain.

### Analysis of the transcription of selected genes by quantitative RT-PCR

**Fig. 4:**
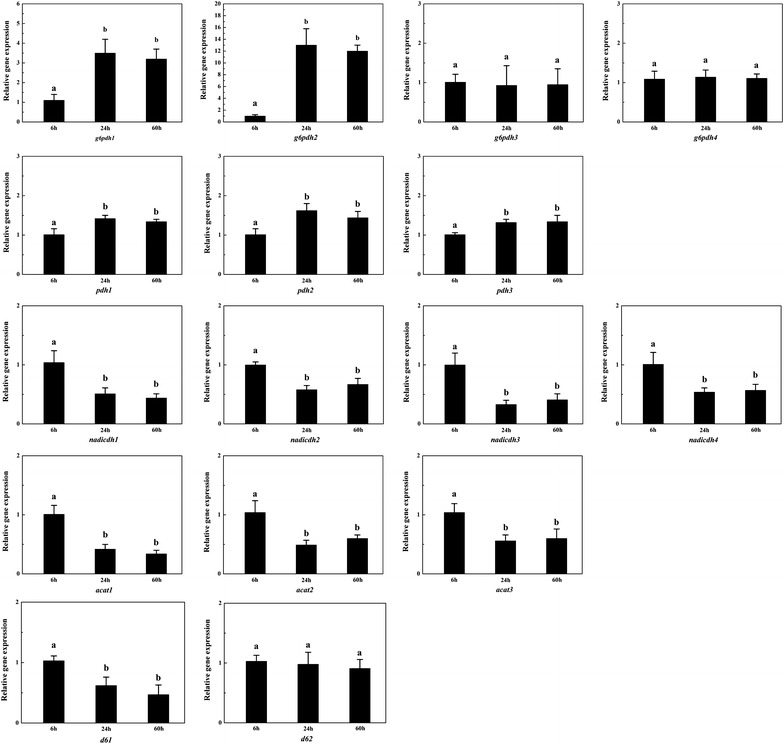
RT-PCR analysis of the transcription level of selected genes in *M. circinellodies* WJ11. Glucose-6-phosphate dehydrogenase was encoded by *g6pdh1*, *2*, *3* and *4*; pyruvate dehydrogenase was encoded by *pdh1*, *2*, and *3*; NAD^+^:isocitrate dehydrogenase was encoded by *nadicdh1*, *2*, *3* and *4*; acetyl-CoA C-acetyltransferase was encoded by *acat1*, *2*, and *3*; fatty acid delta-6 desaturase was encoded by *d61* and *d62.* Values are mean of three biological replicates. *Error bars* represent the standard error of the mean. Values which do not share common superscripts were significantly different to each other (P < 0.05)

Some differentially expressed proteins that participate in key metabolic reactions related to lipid accumulation during the bioprocess, G6PDH, PDH, NAD^+^:ICDH and acetyl-CoA C-acetyltransferase (ACAT), were selected to determine the transcription levels of their genes by quantitative RT-PCR. These proteins are encoded by more than one gene and the mRNA expression profiles of these genes are shown in Fig. 4. G6PDH is encoded by four genes and the transcription levels of *g6pdh1* and *g6pdh2* were significantly higher at 24 h and 60 h of growth (lipid accumulation phase) than at 6 h of growth (balanced growth phase), which is in accordance with the protein expression level in this study and the enzymatic activity in *M. circinellodies* of our previous research [6]. Therefore the *g6pdh1* and *g6pdh2* might play an important role (e.g., supply of NADPH for fatty acids) in lipid accumulation. Unlike *g6pdh1* and *g6pdh2*, the transcription levels of *g6pdh3* and *g6pdh4* were stable during the whole bioprocess.

The mRNA of the genes encoding PDH were increased upon N deficiency. This is coincident with expression level of the protein, suggesting that conversion of pyruvate to acetyl CoA for TCA cycle was increased upon N depletion. Transcription levels of the genes encoding NAD^+^:ICDH were decreased upon N depletion indicating that TCA cycle is retarded after N exhaustion. Thus, the combined coordinated regulation of PDH and NAD^+^:ICDH upon N depletion results in an increased cytosolic acetyl-CoA production for fatty acid biosynthesis. Furthermore, the mRNA of the genes encoding ACAT were also decreased upon N limitation, which is in according with its protein level. This will direct the flux of acetyl-CoA into fatty acid biosynthesis rather than into isoprenoid biosynthesis. Taken together, the quantitative RT-PCR analysis demonstrated the transcription level of some proteins related to lipid accumulation under N deficiency is consistant with their protein level from proteomic data.

*M. circinelloides* is a GLA-producing filamentous fungus and fatty acid delta-6 desaturase converts linoleic acid into GLA, which is an key enzyme for GLA biosynthesis [49]. Thus we also investigated transcription levels of the genes (*d61* and *d62*) encoding fatty acid delta-6 desaturase (Fig. 4). The result showed the transcription level of *d61* was significantly lower at 24 h and 60 h than that at 6 h, while the level of *d62* had no obvious changes in whole bioprocess. The fatty acid composition showed the GLA content in total fatty acid at 24 h and 60 h were both less than half of that at 6 h (Additional file 1: Table S1), which was in according with the transcription level of *d61*.

## Conclusion

This study represents a proteomic analysis of lipid accumulation in *M. circinelloides* WJ11, a higher lipid-producing strain (36 % lipid, w/w), grown under N limited condition. N limitation increased the expression of the proteins involved in ammonia assimilation for the supply of cellular nitrogen but decreased the expression of proteins involved in the biosynthesis of amino acids. Carbon metabolism is crucial for lipid accumulation, which was significantly affected upon N deficiency. Upon N deprivation, the glycolysis pathway, which provides pyruvate, a key precursor for acetyl-CoA, was up-regulated, while the activity of the TCA cycle was retarded, thus, leading more carbon flux to fatty acid biosynthesis. Moreover, there is some evidence that the PPP was up-regulated and then this would increase the production of NADPH needed for fatty acid biosynthesis. Together, coordinated regulation of central carbon metabolism upon N limitation, provide more carbon flux to acetyl-CoA and NADPH for fatty acid biosynthesis. In addition, protein and nucleic acid metabolism was down-regulated upon N limitation, this shifts the cellular metabolism to lipid biosynthesis. Also, some protein involved in energy metabolism, signal transduction, molecular chaperone and redox homeostasis were up-regulated in response to stress condition of N deficiency.

## Methods

### Microorganism and cultivation

*M. circinelloides* WJ11 isolated in our laboratory from soil at Jiangnan University was used in this study [6, 49]. 100 µl spore suspension (approx. 10^7^ spores/mL) of *M. circinelloides* WJ11 was cultivated in 150 mL K & R medium [38] held in 1 L flasks equipped with baffles for 24 h with shaking at 150 rpm and 30 °C, and then used at 10 % (v/v) to inoculate 2 L fermenters containing 1.5 L modified K & R medium (80 g glucose and 2 g diammonium tartrate per liter). Fermenters were controlled at 30 °C with stirring at 700 rpm and aeration at 0.5 v/v min^−1^. The pH was maintained at 6.0 by auto-addition of 4 M KOH or 2 M H_2_SO_4_.

### Analysis of cell dry weight (CDW), culture supernatant and lipid accumulation

Biomass was periodically harvested by filtration using a dried and pre-weighed filter paper and a Buchner funnel under vacuum and washed three times with distilled water, frozen overnight at −80 °C and then freeze-dried. The weight of dry cells was determined gravimetrically. Glucose concentration in the culture medium was measured using a glucose oxidase kit according to the manufacturer’s instructions. Ammonium concentration in the culture filtrate was determined using the indophenol test [50]. Total lipid was extracted and analyzed by the procedure reported in our previous work [6].

### Protein extraction for two-dimensional electrophoresis (2-DE)

Cells taken from the balanced phase of growth (at 6 h), the fast lipid accumulation stage (at 24 h) and the stable stage of lipid accumulation (at 60 h) were filtered as above and washed with cold distilled water at 4 °C. The collected mycelia were flash-frozen in liquid N_2_ and stored at −80 °C. For each condition, protein extraction was performed according to a modified version of the method of Liu et al. [51] and Chen et al. [52]. The frozen mycelia were ground in liquid N_2_, resuspended in cold 10 % (w/v) trichloracetic acid/acetone and allowed to precipitate at −20 °C for 1 h. The samples were centrifuged at 15,000*g* for 15 min at 4 °C and the supernatant was discarded. The pellets were resuspended in cold acetone, kept at −20 °C for 1 h and then centrifuged at 15,000*g* for 15 min at 4 °C. This procedure was repeated twice. The pellets were freeze-dried, suspended in approx. 10 mL extraction buffer [0.7 M sucrose, 0.1 M NaCl, 0.5 M Tris/HCl (pH 7.5), 50 mM EDTA and 0.2 % DTT], mixed with an equal volume of Tris/saturated phenol (1 g/100 mL) (pH 7.5) and then homogenized for 30 min at 4 °C. The homogenate was centrifuged at 5000 *g* for 10 min at 4 °C to collect the phenol phase and the phenol extraction was repeated three time. The combined phenol phases were mixed with five volumes of a precipitation buffer (0.1 M ammonium acetate in methanol). Precipitation was carried out at −20 °C for 1 h and the pellets were washed three times with cold methanol followed by rinsing three time with cold acetone. The protein pellets were dissolved in a lysis buffer [9 M urea, 4 % CHAPS, 1 % immobilized pH gradient buffer (pH 3–10) and 1 % DTT] and then centrifuged at 15,000*g* for 15 min. The supernatants were collected and protein concentrations were determined according to the Bradford method with BSA as a standard.

### 2-DE

Before 2-DE, 1200 µg protein solution was mixed with rehydration buffer [9 M urea, 4 % CHAPS, 1 % immobilized pH gradient buffer (pH 3–10), 1 % DTT and 0.002 % Bromphenol Blue] and then loaded onto the IPG strip (pH 3–10, nonlinear, 24 cm). Strips were focused on a IPGhor Isoelectric Focusing System (GE Healthcare) at 20 °C with the following program: 50 V for 12 h (for rehydration), 100 V for 1 h, 200 V for 1 h, 500 V for 1 h, 1000 V for 1 h, gradient from 1000 to 10,000 V within 1 h and 10,000 V for 11 h. After that, strips were equilibrated at room temperature in two steps: 15 min in equilibration buffer [50 mM Tris/HCl (pH 8.8), 6 M urea, 30 % w/v glycerol and 2 % w/v SDS] with the addition of 1 % DTT followed by 15 min in equilibration buffer with the addition of 2.5 % (w/v) iodoacetamide. The equilibrated strips were transferred onto 12 % SDS-PAGE for the second dimension electrophoresis by using DALT-SIX SDS-PAGE Vertical System (GE Healthcare) at 15 °C with two steps: 100 V for 45 min and 200 V until the Bromophenol Blue reached the bottom of the gel. The gels were fixed with 10 % (w/v) trichloroacetic acid and stained with Coomassie Brilliant Blue G-250.

### Image analysis

Nine 2-DE gels (three independent analytical replicate gels for each growth stage) were scanned at 300 dpi using Image Scanner LabScan (GE Healthcare). Spot detection, gel matching and group analysis of the gels were performed using PDQuest 8.0 software (Bio-Rad). Quantitative analyses were carried out after normalizing the quantities of spots in all gels in order to compensate for non-expression related variations, and quantity of each spot was normalized by total valid spot intensity. For each spot, the mean quantity was computed at every stage, and the spots showing a mean value that changed more than 1.5-fold or less than 0.67-fold (P < 0.05) in different stages were considered differentially expressed proteins.

### Protein identification and database search

Protein spots with different expression levels were manually excised from the gels, washed with Millipore pure water for three times, destained three times with 100 mM NH_4_HCO_3_ in 30 % (v/v) acetonitrile, and then vacuum dried. Every protein spot was digested overnight with 50 ng trypsin (Promega) in 30 μl 25 mM NH_4_HCO_3_ containing 10 % (v/v) acetonitrile at 37 °C. The supernatants were transferred into another tube followed by vacuum dried. The dried peptides were dissolved in 0.1 % trifluoroacetic acid and mixed with an equal volume of 0.7 mg α-cyano-4-hydroxy-*trans*-cinnamic acid/mL in acetonitrile/trifluoroacetic acid (85:0.1 v/v), and then spotted on the sample target plate for analysis using UltrafleXtrem MALDI-TOF/TOF mass spectrometer (Bruker-Daltonics). Tryptic peptides were analyzed in the positive ion reflector mode, and spectra were calibrated using Bruker peptide calibration standard II (Bruker-Daltonics). At least 10 peptide fragments were selected to be analyzed in lift mode. Then spectra were processed by FlexAnalysis software and analyzed by BioTools software (Bruker-Daltonics). An in-house Mascot server (http://www.matrixscience.com) was used for database search and the following parameters were used in the search: NCBInr fungi database; trypsin as the digestion enzyme; monoisotopic peptide values; a maximum of one missed cleavage per peptide; fragment mass tolerance of 0.99 Da and peptide mass tolerance of 300 ppm, together with the acceptance of cysteine carbamidomethylation (fixed modifications) and methionine oxidation (variable modifications). For a positive identification, a score calculated by the Mowse scoring algorithm in MASCOT was considered as significant (P < 0.05).

### Quantitative RT-PCR

Quantitative RT-PCR analysis was performed to quantify the transcriptional levels of genes. Total RNA was extracted from samples taken at the three growth stages with TRIzol and then converted to cDNA using a PrimeScrip 1st strand cDNA Synthesis Kit (Takara) according to the manufacturer’s instructions. The quantitative RT-PCR reaction was performed with CFX Connect Real-Time System (Bio-Rad) and iTaq Universal SYBR Green PCR Supermix (Bio-Rad) was used to identify mRNA level. The primer pairs are listed in Additional file 2: Table S2 using the 18S rRNA of *M. circinelloides* WJ11 as an internal control in PCR amplification.

### Statistical analysis

The mean values and the standard error of the mean were calculated from the data obtained from three biological replicates. A statistical analysis of the obtained data was carried out using SPSS 16.0 for Windows (SPSS Inc., Chicago, IL). One way analysis of variance (ANOVA) with Tukey’s test was conducted on the data, and P < 0.05 was considered significantly different.

## Additional files

10.1186/s12934-016-0428-4 Fatty acid composition and GLA production in *M. circinelloides* WJ11 at 6 h, 24 h and 60 h.
10.1186/s12934-016-0428-4 Primer sequences used for qRT-PCR.

## Abbreviations

N: nitrogen
CDW: cell dry weight
PUFAs: polyunsaturated fatty acids
GLA: γ-linolenic acid
ACL, ATP: citrate lyase
TFAs: total fatty acids
GS: glutamine synthase
BCAA: branched-chain amino acids
SAS: *S*-adenosylmethionine synthase
ROS: reactive oxygen species
FBA: fructose-bisphosphate aldolase
G3P: glyceraldehyde 3-phosphate
DHAP: dihydroxyacetone phosphate
PDH: pyruvate dehydrogenase
PGM: phosphoglycerate mutase
3PGA: 3-phosphoglycerate
2PGA: 2-phosphoglycerate
PPP: pentose phosphate pathway
ME: malic enzyme
G6PDH: glucose-6-phosphate dehydrogenase
NAD^+^:ICDH: NAD^+^:isocitrate dehydrogenase
ALDH: aldehyde dehydrogenase
ACS: acetyl-CoA synthase
ACAT: acetyl-CoA C-acetyltransferase
TBE: thiazole biosynthetic enzyme
HSPs: heat shock proteins
Prx: peroxiredoxin
GPx: glutathione peroxidase

## References

[1] Thorpe R, Ratledge C (1972). Fatty acid distribution in triglycerides of yeasts grown on glucose or n-alkanes. J Gen Microbiol.

[2] Ratledge C (2014). The role of malic enzyme as the provider of NADPH in oleaginous microorganisms: a reappraisal and unsolved problems. Biotech Lett.

[3] Ratledge C, Wynn JP (2002). The biochemistry and molecular biology of lipid accumulation in oleaginous microorganisms. Adv Appl Microbiol.

[4] Ratledge C, Akoh CC (2005). Microbial production of gamma-linolenic acid. Handbook of functional lipids.

[5] Botham PA, Ratledge C (1979). A biochemical explanation for lipid accumulation in *Candida* 107 and other oleaginous micro-organisms. J Gen Microbiol.

[6] Tang X, Chen H, Chen YQ, Chen W, Garre V, Song Y, Ratledge C (2015). Comparison of biochemical activities between high and low lipid-producing strains of *Mucor circinelloides*: an explanation for the high oleaginicity of strain WJ11. PLoS ONE.

[7] Boulton CA, Ratledge C (1981). Correlation of lipid accumulation in yeasts with possession of ATP: citrate lyase. J Gen Microbiol.

[8] Tamano K, Bruno KS, Karagiosis SA, Culley DE, Deng S, Collett JR, Umemura M, Koike H, Baker SE, Machida M (2013). Increased production of fatty acids and triglycerides in *Aspergillus oryzae* by enhancing expressions of fatty acid synthesis-related genes. Appl Microbiol Biotechnol.

[9] Zhang H, Zhang L, Chen H, Chen YQ, Chen W, Song Y, Ratledge C (2014). Enhanced lipid accumulation in the yeast *Yarrowia lipolytica* by over-expression of ATP: citrate lyase from Mus musculus. J Biotechnol.

[10] Wynn JP, bin Abdul Hamid A, Ratledge C (1999). The role of malic enzyme in the regulation of lipid accumulation in filamentous fungi. Microbiology.

[11] Wynn JP, Ratledge C (1997). Malic enzyme is a major source of NADPH for lipid accumulation by *Aspergillus nidulans*. Microbiology.

[12] Zhu Z, Zhang S, Liu H, Shen H, Lin X, Yang F, Zhou YJ, Jin G, Ye M, Zou H (2012). A multi-omic map of the lipid-producing yeast *Rhodosporidium toruloides*. Nat Commun.

[13] Liu Z, Gao Y, Chen J, Imanaka T, Bao J, Hua Q (2013). Analysis of metabolic fluxes for better understanding of mechanisms related to lipid accumulation in oleaginous yeast *Trichosporon cutaneum*. Bioresour Technol.

[14] Paula Alonso A, Dale VL, Shachar Hill Y (2010). Understanding fatty acid synthesis in developing maize embryos using metabolic flux analysis. Metab Eng.

[15] Xiong W, Liu L, Wu C, Yang C, Wu Q (2010). 13C-tracer and gas chromatography-mass spectrometry analyses reveal metabolic flux distribution in the oleaginous microalga *Chlorella protothecoides*. Plant Physiol.

[16] Wasylenko TM, Ahn WS, Stephanopoulos G (2015). The oxidative pentose phosphate pathway is the primary source of NADPH for lipid overproduction from glucose in *Yarrowia lipolytica*. Metab Eng.

[17] Zhao L, Zhang H, Wang L, Chen H, Chen YQ, Chen W, Song Y (2015). 13 C-metabolic flux analysis of lipid accumulation in the oleaginous fungus *Mucor circinelloides*. Bioresour Technol.

[18] Yang ZK, Ma YH, Zheng JW, Yang WD, Liu JS, Li HY (2014). Proteomics to reveal metabolic network shifts towards lipid accumulation following nitrogen deprivation in the diatom *Phaeodactylum tricornutum*. J Appl Phycol.

[19] Garnier M, Carrier G, Rogniaux H, Nicolau E, Bougaran G, Saint-Jean B, Cadoret J-P (2014). Comparative proteomics reveals proteins impacted by nitrogen deprivation in wild-type and high lipid-accumulating mutant strains of *Tisochrysis lutea*. J Proteomics.

[20] Song P, Li L, Liu J (2013). Proteomic analysis in nitrogen-deprived *Isochrysis galbana* during lipid accumulation. PLoS ONE.

[21] Liu H, Zhao X, Wang F, Li Y, Jiang X, Ye M, Zhao ZK, Zou H (2009). Comparative proteomic analysis of *Rhodosporidium toruloides* during lipid accumulation. Yeast.

[22] Chen H, Hao G, Wang L, Wang H, Gu Z, Liu L, Zhang H, Chen W, Chen YQ (2015). Identification of a critical determinant that enables efficient fatty acid synthesis in oleaginous fungi. Sci Rep.

[23] Xia C, Zhang J, Zhang W, Hu B (2011). A new cultivation method for microbial oil production: cell pelletization and lipid accumulation by *Mucor circinelloides*. Biotechnol Biofuels.

[24] Tang X, Zhang H, Chen H, Chen YQ, Chen W, Song Y (2014). Effects of 20 standard amino acids on the growth, total fatty acids production, and γ-linolenic acid yield in *Mucor circinelloides*. Curr Microbiol.

[25] Stacey G, Van Baalen C, Tabita FR (1979). Nitrogen and ammonia assimilation in the cyanobacteria: regulation of glutamine synthetase. Arch Biochem Biophys.

[26] Magasanik B, Kaiser CA (2002). Nitrogen regulation in *Saccharomyces cerevisiae*. Gene.

[27] Chipman D, Ze Barak, Schloss JV (1998). Biosynthesis of 2-aceto-2-hydroxy acids: acetolactate synthases and acetohydroxyacid synthases. BBA Protein Struct Mol Enzymol.

[28] Myers JW (1961). Dihydroxy acid dehydrase: an enzyme involved in the biosynthesis of isoleucine and valine. J Biol Chem.

[29] Chunduru SK, Mrachko GT, Calvo K (1989). Mechanism of ketol acid reductoisomerase. Steady-state analysis and metal ion requirement. Biochemistry.

[30] Ichihara A, Koyama E (1966). Transaminase of branched chain amino acids. J Biochem.

[31] Huber TA, Streeter JG (1984). Asparagine biosynthesis in soybean nodules. Plant Physiol.

[32] Fujioka M, Nakatani Y (1972). Saccharopine dehydrogenase. Eur J Biochem.

[33] Miyazaki J, Kobashi N, Nishiyama M, Yamane H (2003). Characterization of homoisocitrate dehydrogenase involved in lysine biosynthesis of an extremely thermophilic bacterium, *Thermus thermophilus* HB27, and evolutionary implication of β-decarboxylating dehydrogenase. J Biol Chem.

[34] Kikuchi G (1973). The glycine cleavage system: composition, reaction mechanism, and physiological significance. Mol Cell Biochem.

[35] Lu SC (2000). S-adenosylmethionine. Int J Biochem cell B.

[36] Shin R, Berg RH, Schachtman DP (2005). Reactive oxygen species and root hairs in Arabidopsis root response to nitrogen, phosphorus and potassium deficiency. Plant Cell Physiol.

[37] Guy C, Carter J (1982). Effect of low temperature on the glutathione status of plant cells. Plant Cold Hardiness Freez Stress.

[38] Kendrick A, Ratledge C (1992). Desaturation of polyunsaturated fatty acids in Mucor circinelloides and the involvement of a novel membrane-bound malic enzyme. Eur J Biochem.

[39] Marsh JJ, Lebherz HG (1992). Fructose-bisphosphate aldolases: an evolutionary history. Trends Biochem Sci.

[40] Song Y, Wynn JP, Li Y, Grantham D, Ratledge C (2001). A pre-genetic study of the isoforms of malic enzyme associated with lipid accumulation in Mucor circinelloides. Microbiology.

[41] Zhang Y, Adams IP, Ratledge C (2007). Malic enzyme: the controlling activity for lipid production? Overexpression of malic enzyme in *Mucor circinelloides* leads to a 2.5-fold increase in lipid accumulation. Microbiology.

[42] Noctor G, Mhamdi A, Chaouch S, Han Y, Neukermans J, Marquez-Garcia B, Queval G, Foyer CH (2012). Glutathione in plants: an integrated overview. Plant, Cell Environ.

[43] Machado C, Oliveira RCL, Boiteux S, Praekelt UM, Meacock PA, Menck CFM (1996). Thi1, a thiamine biosynthetic gene in Arabidopsis thaliana, complements bacterial defects in DNA repair. Plant Mol Biol.

[44] Machado CR, Praekelt UM, Oliveira RCL, Barbosa ACC, Byrne KL, Meacock PA, Menck CF (1997). Dual role for the yeast THI4 gene in thiamine biosynthesis and DNA damage tolerance. J Mol Biol.

[45] Wynn JP, Hamid AA, Li Y, Ratledge C (2001). Biochemical events leading to the diversion of carbon into storage lipids in the oleaginous fungi *Mucor circinelloides* and *Mortierella alpina*. Microbiology.

[46] Hermeking H, Benzinger A (2006). 14-3-3 proteins in cell cycle regulation. Semin Cancer Biol.

[47] Choi YE, Kwon KW, Lee JC, Woo SY (2007). Expression of the rice cytoplasmic cysteine synthase gene in tobacco reduces ozone-induced damage. Plant Biotechnol Rep.

[48] Bell A, Monaghan P, Page AP (2006). Peptidyl-prolyl cis–trans isomerases (immunophilins) and their roles in parasite biochemistry, host-parasite interaction and antiparasitic drug action. Int J Parasitol.

[49] Tang X, Zhao L, Chen H, Chen YQ, Chen W, Song Y, Ratledge C (2015). Complete genome sequence of a high lipid-producing strain of *Mucor circinelloides* WJ11 and comparative genome analysis with a low lipid-producing strain CBS 277.49. PLoS ONE.

[50] Chaney AL, Marbach EP (1962). Modified reagents for determination of urea and ammonia. Clin Chem.

[51] Liu X, Wu H, Ji C, Wei L, Zhao J, Yu J (2013). An integrated proteomic and metabolomic study on the chronic effects of mercury in Suaeda salsa under an environmentally relevant salinity. PLoS ONE.

[52] Chen Y, Pang Q, Dai S, Wang Y, Chen S, Yan X (2011). Proteomic identification of differentially expressed proteins in Arabidopsis in response to methyl jasmonate. J Plant Physiol.

