# Supplementary material for: Proteomics analysis of high lipid-producing strain Mucor circinelloides WJ11: an explanation for the mechanism of lipid accumulation at the proteomic level
Source: Microb Cell Fact. 2016 Feb 11;15:35. doi: 10.1186/s12934-016-0428-4 (PMC4750200; doi:10.1186/s12934-016-0428-4)
Supplement: Supplementary file 1 — 10.1186/s12934-016-0428-4 Fatty acid composition and GLA production in M. circinelloides WJ11 at 6 h, 24 h and 60 h. [file 12934_2016_428_MOESM1_ESM.docx]

**Supplementary material**

**Table S1 Fatty acid composition and GLA production in *M. circinelloides* WJ11 at 6 h, 24 h and 60 h.**

| Time (h) | Fatty acid composition (%, w/w of total fatty acids) | | | | | | | | GLA production  (%, w/w of cell dry weight) |
| --- | --- | --- | --- | --- | --- | --- | --- | --- | --- |
|  | 14:0 | 16:0 | 16:1 | 18:0 | 18:1 | 18:2 | 18:3  (GLA) | Others |  |
| 6 | ND | 20.7±0.3 | ND | 3.8±0.2 | 25.6±0.2 | 19.6±0.1 | 30.3±1.1 | ND | 1.6±0.1 |
| 24 | 1.1±0 | 23.4±0.4 | 0.7±0 | 7.2±0.7 | 34.6±0.2 | 15.2±0.3 | 14.9±0.5 | 2.9±0 | 3.1±0.1 |
| 60 | 1.2±0 | 23.7±0.1 | 0.8±0.1 | 7.0±0.6 | 37.3±0.9 | 14.7±0.2 | 13.0±0.1 | 2.3±0.7 | 4.3±0 |
