# Supplementary material for: Proteomics analysis of high lipid-producing strain Mucor circinelloides WJ11: an explanation for the mechanism of lipid accumulation at the proteomic level
Source: Microb Cell Fact. 2016 Feb 11;15:35. doi: 10.1186/s12934-016-0428-4 (PMC4750200; doi:10.1186/s12934-016-0428-4)
Supplement: Supplementary file 2 — 10.1186/s12934-016-0428-4 Primer sequences used for qRT-PCR. [file 12934_2016_428_MOESM2_ESM.docx]

**Supplementary material**

**Table S2 Primer sequences used for qRT-PCR.**

| Primer Product | Sequence(5′-3′) | Length (bp) |
| --- | --- | --- |
| *g6pdh1-F* | CGACAACCGCATGAAAGGTG | 160 |
| *g6pdh1-R* | ACAGTCCATCTTGGTGCGAG |  |
| *g6pdh2-F* | CCATGGAGCGTCCCATTTCT | 159 |
| *g6pdh2-R* | TAGGCACGCTCTCATCATCG |  |
| *g6pdh3-F* | GCCCATCGCTATTGAGGACA | 154 |
| *g6pdh3-R* | ACCTTCCCAACGCTCATTGT |  |
| *g6pdh4-F* | CAAGAACGCTGCCCGTAATG | 150 |
| *g6pdh4-R* | GCCAAGTCAGAGTAACGGCT |  |
| *pdh1-F* | GGCCGAATTGATGGGTCGTA | 122 |
| *pdh1-R* | CGGCACCAAGAGGAACTTGA |  |
| *pdh2-F* | CCGTTCCTGGTCTCAAGGTT | 194 |
| *pdh2-R* | GCGCTCAATCTTGGCCTTAC |  |
| *pdh3-F* | GCCGTAGCTGTTGGTATGGA | 164 |
| *pdh3-R* | ATGGAGCCACCTTTGCCTTT |  |
| *nadicdh1-F* | ACTACCGCCACTCCCAGTAT | 150 |
| *nadicdh1-R* | AATAGGAGTCTTTGCGGCGG |  |
| *nadicdh2-F* | ACTTTGGTGCCTGGAGATGG | 145 |
| *nadicdh2-R* | ACGTGTCCTGCATCAAACCA |  |
| *nadicdh3-F* | ACGACGTCTCTGGTTTGACC | 171 |
| *nadicdh3-R* | GAGAGGCGTACATGTCGAGG |  |
| *nadicdh4-F* | TCCCTGGTGATGGTATCGGT | 161 |
| *nadicdh4* | CTTGTTGCGACGGAGAGACT |  |
| *acat1-F* | CGTGGTAAGCCTGATCGTGT | 122 |
| *acat1-R* | GAGTTGGGAGCAGTAACGGA |  |
| *acat2-F* | AAGGAAGGTACTGTCACGGC | 187 |
| *acat2-R* | CCTTGGGTAAGGCAAGAGCA |  |
| *acat3-F* | GGTTGCCCTGAAACTACCGA | 180 |
| *acat3-R* | ATGACCATAGGCAGCACCAC |  |
| *d61-F* | GAGCACCATGTCTTCCCCAA | 119 |
| *d61-R* | GTGCCCTTCCAGAAACCAGT |  |
| *d62-F* | ACTGGTTACTTGCTCGCCAT | 115 |
| *d62-R* | CGCGACCAGTGATACATTGC |  |
| 18S RNA*-F* | GTCTTAGATGAGGTGGCCTGG | 105 |
| 18S RNA*-R* | TGGCTAGAGACTATAAACAACCG |  |
